# Suizidale Gasembolie im Krankenhaus

**DOI:** 10.1007/s00194-021-00528-y

**Published:** 2021-09-14

**Authors:** A. Böckers, S. Steinhoff, T. Scholl, Sebastian N. Kunz

**Affiliations:** 1 Institut für Rechtsmedizin, Universitätsklinikum Ulm, Prittwitzstr. 6, 89075 Ulm, Deutschland; 2 Universität Ulm, Ulm, Deutschland; 3 Radiologie, Bundeswehrkrankenhaus Ulm, Ulm, Deutschland; 4 Pathologie, Bundeswehrkrankenhaus Ulm, Ulm, Deutschland

**Keywords:** Forensische Radiologie, Todesursache, Luftembolie, Suizid, Postmortale Computertomographie, Forensic radiology, Cause of death, Air embolism, Suicide, Postmortem computer tomography

## Abstract

Luftembolien sind im klinischen Alltag nach traumatischen oder iatrogenen Ereignissen eine häufig zu beobachtende Entität. Fälle einer in suizidaler Absicht herbeigeführten Luftembolie sind selten. Die Konnektivität von luft- und flüssigkeitsführenden Schlauchsystemen ermöglicht die Zufuhr großer Gasmengen in kurzer Zeit mit häufig tödlichem Ausgang. Der Einsatz einer Computertomographie vor der Obduktion ist in solchen Fällen obligat und ermöglicht eine umfassende Darstellung der zugeführten Gasmengen. Wir präsentieren den ungewöhnlichen Fall einer suizidalen venösen Gaszufuhr mittels eines stationären Sauerstoffgerätes in einem Krankenhaus.

## Abstract (en)

Air embolisms are a common entity seen in clinical practice after traumatic or iatrogenic events. Cases of a suicidally induced air embolism are rare. The connectivity of air-carrying and fluid-carrying tubing systems allow large gas volumes to be infused in a short period of time, usually with fatal outcome. In such cases, the use of computed tomography prior to autopsy is mandatory and provides a comprehensive visualization of introduced gas volumes. We present an unusual case of a suicidal gas embolism, in which a man in hospital connected a tube supplying oxygen to a venous catheter.

## Falldarstellung

### Einleitung

Luftembolien sind eine im klinischen Alltag nach traumatischen Ereignissen oder medizinischen Eingriffen häufig zu beobachtende Entität [1]. Meistens entstehen sie durch das Ansaugen von Luft in eröffnete, klappenlose Venen oberhalb der Herzebene; deutlich seltener werden Luftembolien durch Druckapplikation von Luft in das venöse System hervorgerufen [8]. Luftembolien können asymptomatisch oder symptomatisch verlaufen. Die Folgen einer Luftembolie ergeben sich dabei aus unterschiedlichen Faktoren, wie der Luftmenge, der Schnelligkeit des Lufteintritts, dem Funktionszustand des kardiopulmonalen Systems der betroffenen Person sowie der Embolielokalisation im Gefäßsystem [1]. In der überwiegenden Anzahl der Fälle treten Luftembolien als Nebenbefund auf, haben aber auch in Abhängigkeit von den oben genannten Faktoren eine todesursächliche Bedeutung.

### Klinischer Verlauf

#### Anamnese

Ein 74-jähriger übergewichtiger Patient (Body Mass Index 39,9 kg/m^2^) wurde mit Verdacht auf eine Urosepsis stationär aufgenommen. Zwei Tage zuvor wurde ein Tumor-Stent-Wechsel bei bestehender Harnleiterstenose rechts vorgenommen. Ursächlich für die Stenose war eine vor 10 Jahren durchgeführte subtotale Kolektomie aufgrund eines Karzinoms im Colon transversum. Vorbekannt waren weiterhin ein arterieller Hypertonus, ein insulinpflichtiger Diabetes mellitus Typ 2, Asthma bronchiale sowie eine kardiale Vorbelastung nach biologischem Klappenersatz bei hochgradiger Aortenklappenstenose und eine absolute Vorhofarrhythmie bei Vorhofflimmern. Psychologische Vorerkrankungen oder eine suizidale Vorgeschichte waren nicht bekannt.

#### Klinischer Befund

Bei Aufnahme war der Patient fiebrig (39 °C) mit einer Atemfrequenz von 35/min und stabilen Vitalparametern (Blutdruck 132/65 mm Hg; Herzfrequenz 101 Schläge/min) mit einer Blutsauerstoffsättigung von 97 % unter nasaler Sauerstoffgabe (2 l/min). Der Bewusstseinsgrad wurde zwar auf der Glasgow-Koma-Skala mit 15 bewertet, allerdings machte der Patient zeitweise einen somnolenten und desorientierten Eindruck.

Laborchemisch ergaben sich neben einem Blutzuckerspiegel von 251 mg/dl und einer Hypokaliämie eine typische Infektkonstellation sowie eine nitritnegative Leukozyt- und Hämaturie im Urinstatus. Eine SARS-CoV-2-Infektion wurde ausgeschlossen.

#### Diagnose

Die Verdachtsdiagnose einer Urosepsis wurde schließlich durch eine mikrobiologische Untersuchung in Blutkulturen des Patienten (*Escherichia-coli*-Nachweis) gesichert.

#### Therapie und Verlauf

Zur weiteren Therapie erhielt der Patient einen periphervenösen Zugang in die rechte Ellenbeuge zur i.v.-Flüssigkeitszufuhr (1000 ml Jonosteril/Tag) und zur antibiotischen Behandlung. Bei bestehender Penicillinallergie wurde eine i.v.-Therapie mit einem Carbapenempräparat begonnen.

**Figure 1:**
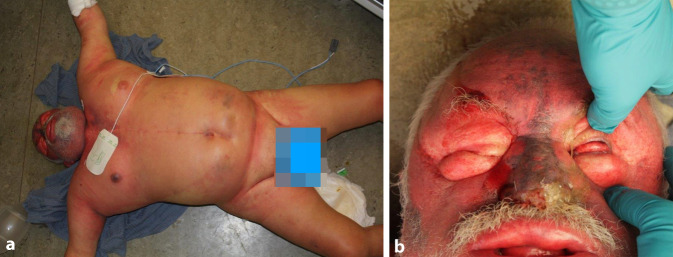

Im Verlauf der folgenden beiden Tage verbesserte sich der Allgemeinzustand des Mannes, und er entfieberte zunehmend. Am Morgen des dritten Tages nach Aufnahme erhielt er um 07:10 Uhr eine neue Infusionsflasche Jonosteril und wurde zu diesem Zeitpunkt als orientiert und nicht auffällig beschrieben. Gegen 08:55 Uhr wurde der Mann von einer Reinigungskraft auf dem Boden liegend vorgefunden (Abb. 1a). Auffallend war eine massive Stauung des Gesichtes mit zugeschwollenen Augen und aufgedunsenen Lippen (Abb. 1a, b). Der Körper des Mannes habe starr und aufgedunsen gewirkt. Da er auf Ansprache keine Reaktion zeigte und keine Atmung festgestellt werden konnte, wurde mit sofortigen kardiopulmonalen Reanimationsmaßnahmen begonnen (09:00 Uhr). Unter dem Verdacht eines anaphylaktischen Schockes wurde die laufende Infusion wenige Minuten später vom periphervenösen Zugang diskonnektiert und dabei festgestellt, dass aus dem Infusionsschlauch Luft ausströmte. Bei schwierigen Beatmungsverhältnissen und fehlender Herztätigkeit wurden die Reanimationsmaßnahmen bei anhaltender Asystolie um 09:30 Uhr beendet. Bei Verdacht auf einen nichtnatürlichen Tod wurden polizeiliche Ermittlungen eingeleitet. Auf Nachfrage beim behandelndem Hausarzt des Verstorbenen wurde ersichtlich, dass dieser bereits früher wiederholt Suizidgedanken geäußert habe.

**Figure 2:**
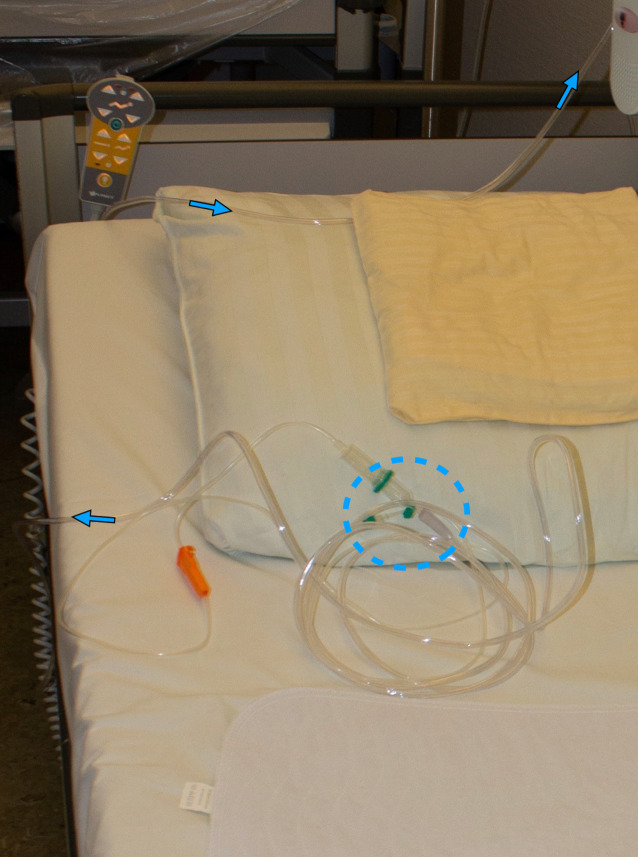

**Figure 3:**
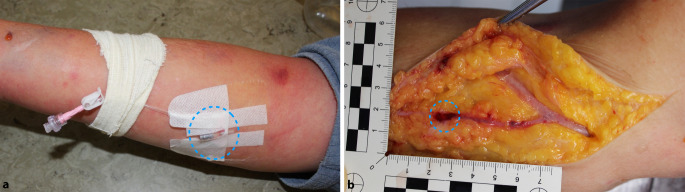

Es konnte polizeitechnischerseits rekonstruiert werden, dass das zur Nasensonde leitende und O_2_-Gas (3 l/min) transportierende Schlauchsystem mit dem Infusionssystem konnektiert und somit O_2_-Gas in das venöse System geleitet worden war (Abb. 2). Eintrittspforte war ein peripherer Gefäßzugang in der rechten Armbeuge (Abb. 3a, b).

### Postmortale Diagnostik

#### Postmortale Computertomographie

Unmittelbar vor Durchführung der Autopsie wurde ca. 72 h nach dem Versterben des Mannes eine postmortale CT-Untersuchung vorgenommen (Fa. Siemens Definition AS [Siemens Healthcare GmbH, Erlangen, Deutschland], Multislice-Spiral CT; Kollimation 128 × 0,6 mm).

**Figure 4:**
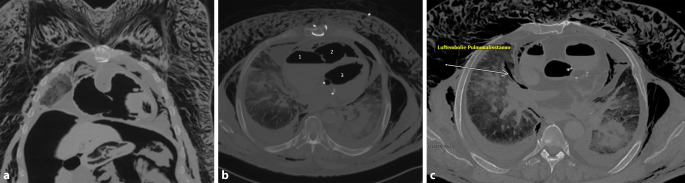

**Figure 5:**
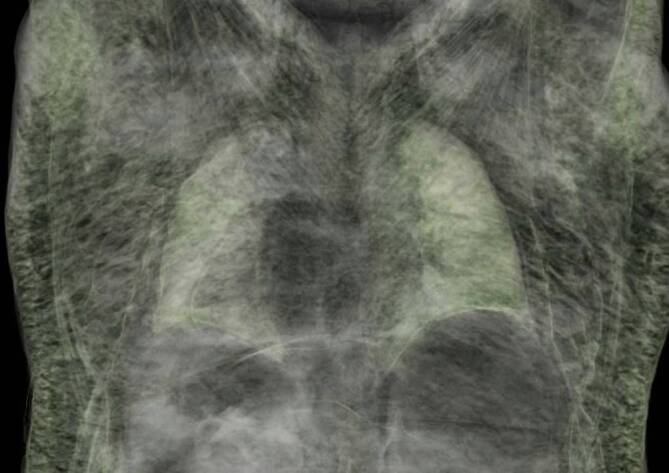

Es zeigte sich ein typischer CT-Befund nach stattgehabter Gasembolie mit gasgefülltem rechtem Vorhof, rechtem und linkem Ventrikel, teilweise Gas bis in die Aorta ascendens und in die ventralen Pulmonalarterien (Abb. 4a–c). Die Untersuchung ergab ebenfalls ein ausgeprägtes mediastinales und kutanes sowie subkutanes Weichteilemphysem mit Punctum maximum thorakal (Abb. 4). Es konnte eine Auffiederung der Muskulatur des Thorax (Mm. pectorales) diagnostiziert werden. Das Weichteil‑/Subkutanemphysem zog sich nach kaudal bis zu den Oberschenkeln hin, bei aufgespanntem Skrotum (Abb. 5). Zusätzlich waren freie Luft intraabdominell bei fehlender Hohlorganperforation (permeative Verteilung) sowie der Nachweis von Bläschen in den venösen Blutleitern des rechten Armes und des Schädels festzustellen.

#### Autoptischer Befund

##### Äußere Besichtigung.

Der Verstorbene zeigte deutliche Stauungszeichen bei allgemein aufgedunsener Körperhülle. Reichlich und kräftig ausgeprägte punktförmige Einblutungen wurden betont in den oberhalb des Herzniveaus gelegenen Körperabschnitten, einschließlich der Lid- und Bindehäute sowie in den Lippenumschlagfalten, festgestellt. Das Weichteilgewebe war, in einer Ausdehnung vom Gesicht bis in die Oberschenkel und die Handrücken reichend, auf Palpation massiv gasblasenunterfüttert, der Hodensack gasgebläht vergrößert. Im Bereich der rechten Ellenbeuge zeigte sich eine von einzelnen intrakutanen Gasbläschen (2–3 mm) umgebene Punktionsstelle. Die Venenverweilkanüle (Vasuflo T®; 1,75 × 51 mm; 16 G; Durchflussrate 196 ml/min) war bereits entfernt worden.

##### Innere Besichtigung.

**Figure 6:**
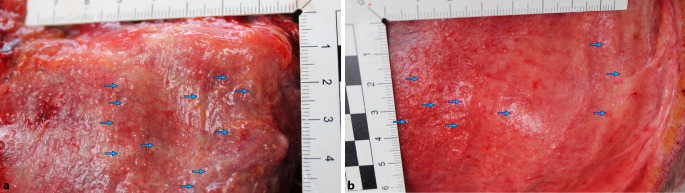

Die Infiltration der Weichteile mit Gasblasen konnte makroskopisch im Thorax- sowie im Hals- und Kopfbereich bestätigt werden (Abb. 4a–c). Dieses Phänomen zeigte sich beispielsweise auch im Bereich des Kehlkopfes (Abb. 6a) und in der Kopfschwarte (Abb. 6b). Nach Eröffnen von Brusthöhle und nachfolgend des Herzbeutels wurde zum Gasnachweis die modifizierte Luftembolieprobe nach Richter [5] durchgeführt. Aus beiden Herzkammern konnten mit einer Kanüle großvolumige Gasansammlungen asserviert und unter Wasser im Herzbeutel in Headspace-Analysegefäße überführt werden. Bei dem mit einer biologischen Aortenklappe versorgten Herzen fanden sich auffällige Zeichen einer chronischen Herzmehrbelastung sowie ein Überschreiten des kritischen Herzgewichtes (740 g), Pleuraergüsse beidseits und eine Endokardfibrose bei mäßiggradig ausgeprägter Koronarsklerose. Das Foramen ovale war verschlossen. Es zeigte sich ein Abdominalsitus im Zustand nach Entfernung des vollständigen Kolonrahmens und Anlage einer Ileum-Sigmoid-Anastomose. Im rechten Harnleiter fand sich ein korrekt eingelegter Doppel-J-Katheter. Die Anlage der Venenverweilkanüle erfolgte in die V. basilica des rechten Arms. Bei Präparation des umliegenden Gewebes der Punktionsstelle waren zahlreiche Gasblasen feststellbar. Im Bereich der Punktionsstelle konnte nach 2,5 cm eine Aussackung mit Einblutung in die Gefäßwand der V. basilica dargestellt werden (Abb. 3b). Das Gefäß war hier jedoch intakt. In unmittelbarer Umgebung zeigte sich eine deutliche Gasblasenunterfütterung des Gewebes.

#### Histologischer Befund

Von dem im Rahmen der Obduktion gewonnenen Gewebsmaterial aus Herz, Lungen, Nieren und Schilddrüse wurden nach Härtung in Formalin kleine Gewebsstückchen zugeschnitten und nach gründlicher Entwässerung in Paraffin eingebettet. Nach Anfertigung von Dünnschnittpräparaten erfolgte die Anfärbung mittels Hämatoxylin-Eosin und mittels der immunhistochemischen Färbung S‑100.

**Figure 7:**
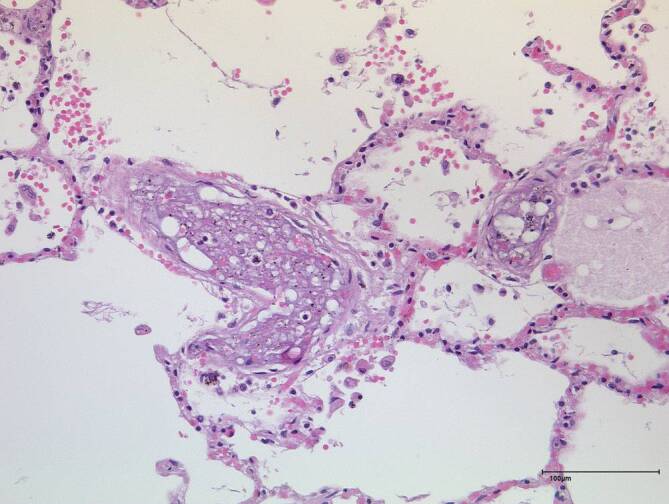

In der Lunge zeigten sich immer wieder kleine Pulmonalarterienäste, deren Lumen mit organisierten Fibrinpräzipitaten verlegt sind, in denen wiederum rundliche Aussparungen zur Darstellung kommen. In der S100-Färbung kann gezeigt werden, dass es sich bei diesen Leerräumen nicht um Fettzellen handelt, und dass somit von einem Einschluss von Luftblasen ausgegangen werden kann (Abb. 7). Auch in den nachgeschalteten Lungenvenen lassen sich teilweise (am ehesten hämostasebedingte) Fibrinpräzipitate nachweisen; in diesen kommen keine Luftblasen zur Darstellung.

Die anderen untersuchten Organe waren unauffällig.

#### Chemisch-toxikologische Zusatzuntersuchung

Aufgrund der eindeutigen morphologischen Befundlage und der zweifelsfreien Ermittlungsergebnisse vor Ort wurde seitens der Staatsanwaltschaft auf eine chemisch-toxikologische Zusatzuntersuchung sowie Bestimmung des asservierten Gases verzichtet.

Zusammenfassend kann davon ausgegangen werden, dass es durch den Anschluss der O_2_-Versorgung an den Gefäßzugang in die rechte V. basilica zu einem relevanten Gaseinstrom in das Gefäßsystem mit Fortleitung zum Herzen sowie in die Lungenarterien gekommen war. Per definitionem wird dies als pulmonale Gasembolie bezeichnet. Dies führte zu einer akuten Widerstandserhöhung im Lungenkreislauf und einem verminderten Blutrückstrom zum linken Vorhof. Es kommt zu einer Mehrbelastung des rechten Herzens, konsekutiven Herzrhythmusstörungen und letztlich zum Tod durch ein akutes Rechtsherzversagen.

### Diskussion

Im vorliegenden Fall ergab sich laut polizeilichen Angaben eine Sauerstoffzufuhr von ca. 3 l/min, was einer Zufuhr von 50 ml/s entspricht. Das lebensbedrohliche Mindestvolumen, welches in den Blutkreislauf eindringen muss, liegt in Abhängigkeit von der Injektionsgeschwindigkeit und vom Zustand des Herz-Kreislauf-Systems bei ca. 3–5 ml/kgKG (70–130 ml) bzw. 300–500 ml/s bei einer Injektionsrate von 100 ml/s [1, 5, 10]. Im aktuellen Fall kann somit davon ausgegangen werden, dass bereits nach wenigen Sekunden eine tödliche Dosis Sauerstoff ins Blutsystem eingebracht worden war, und dass das chronisch vorgeschädigte Herz nur sehr begrenzte Kompensationsmöglichkeiten gehabt hatte. Es ist daher äußerst wahrscheinlich, dass der Mann zum Zeitpunkt der Auffindung bereits nicht mehr reanimationsfähig gewesen war. Beachtlich ist in diesem Fall die große Gasmenge, die sich offensichtlich postmortal im Weichgewebe ausgebreitet hatte. Trotz des kleinvolumigen Gefäßzugangs im Verhältnis zur großvolumigen Sauerstoffzufuhr konnte reichlich Gas sowohl in das Gefäßsystem als auch in die Weichteile transportiert werden. Im Rahmen der postmortalen CT-Untersuchung wurde durch die Gasverteilung geschlossen, dass es zu mehrfacher Ruptur der venösen Gefäße, u. a. im rechten Oberarmbereich, gekommen war.

Ähnliche Fälle im Krankenhaus in suizidaler Absicht erfolgter tödlicher Luftembolien, wie der hier präsentierte Fall, sind selten. Aus rechtsmedizinischer und kriminalistischer Sicht gilt es dabei immer, den Vorwurf eines Behandlungsfehlers oder eines Fremdverschuldens zu prüfen. Selbst nach Ausschluss eines Fremdverschuldens ist nicht immer eindeutig zu klären, ob ein Suizid oder ein Unfallgeschehen vorliegt [2, 9]. Im vorliegenden Fall ist die große Gasverteilung in den Gefäßen und v. a. im Weichgewebe ungewöhnlich, ließ sich aber durch die Gegebenheiten und durch die rechtsmedizinischen und radiologischen Befunderhebungen rekonstruieren. Gasansammlungen im arteriellen Gefäßsystem (z. B. in der Aorta ascendens) wurden in vergleichbaren Fällen einer Gasembolie von anderen Autoren beschrieben [2, 4, 7]. Pathophysiologisch ist dieser Befund durch einen Rechts-links-Shunt erklärbar. Allerdings konnte im aktuellen Fall ein Rechts-links-Shunt durch ein offenes Foramen ovale ausgeschlossen werden. Bunai et al. [2] erklären den Übertritt von Gas ins arterielle Gefäßsystem durch einen erhöhten pulmonalarteriellen Druck, der zur Eröffnung intrapulmonaler arteriovenöser Shunts führt.

Eine Beibringung durch fremde Hand konnte anhand der Spurenlage und nach Auswertung der Zeugenaussagen ausgeschlossen werden. Bei geklärtem Hergang der Ereignisse wurde auf eine Gasanalyse der Asservate verzichtet.

Im klinischen Kontext sollten als besonders gefährdete Gruppe Patient*innen benannt werden, die sowohl mit einer i.v.-Flüssigkeitszufuhr als auch einer O_2_-Gabe therapiert werden. In der Vergangenheit wurde wiederholt von Kasuistiken mit tödlichem Ausgang berichtet, in denen die Zuleitungssysteme von nichtmedizinischen Personen diskonnektiert und bewusst oder versehentlich vertauscht rekonnektiert wurden [3, 4, 6, 9]. Auch wenn es sich hier um Einzelfälle handelt, sollte neben einer professionellen Schulung des Pflegepersonals auch eine Anpassung der Zuleitungssysteme z. B. durch eindeutige Kennzeichnungen, die ein Fehlkonnektieren verhindern, angestrebt werden.

Für die rechtsmedizinische Begutachtung ist der sichere Nachweis einer Luftembolie und deren Abgrenzung von Fäulnisgasen von besonderer Bedeutung. Die autoptische Untersuchung eines Verstorbenen erlaubt je nach aufgenommener Gasmenge schon durch Inspektion, Palpation der Gewebe, durch die Nachweismethode nach Richter oder durch histologische Gewebeuntersuchungen die Diagnose einer Luftembolie. Eine Bestimmung der Gasart (z. B. Luft, O_2_, CO_2_, Methan) ist durch weiterführende toxikologische Untersuchungen möglich [3, 7]. Heutzutage wird die frühzeitige (< 24 h) postmortale CT-Untersuchung bei Verdacht auf Lungenembolie empfohlen [1] bzw. sogar als Untersuchungsmethode der Wahl gesehen [6]. So ermöglichen CT-Aufnahmen außerdem die Darstellung von auch geringen Gasansammlung im arteriellen System, wie im Circulus arteriosus Willisii [4] oder der Ganzkörperverteilung von Gasansammlungen im Weichgewebe anhand einer 3D-Rekonstruktion [9]. Zusätzlich ist eine CT-gesteuerte Gasprobenentnahme zur weiterführenden toxikologisch-biochemischen Analyse möglich [3].

Bei Verdachtsfällen, insbesondere nach Durchführung ärztlicher Maßnahmen (z. B. Kraniotomie, ZVK-Anlage, Endoskopie etc. [1]) sollte umgehend eine CT-Untersuchung erfolgen, um eine umfassende Darstellung der aufgenommenen Gasmengen im gesamten Körper zu dokumentieren.

## Fazit für die Praxis

- Eine eindeutige Kennzeichnung und Anpassung der Schlauchsysteme ist im Krankenhausalltag essentiell, um mögliche akzidentelle oder in suizidaler Absicht erfolgte Fehlkonnexionen zu vermeiden.
- Bei Verdacht auf eine Luftembolie ist eine CT-Untersuchung als unverzichtbar anzusehen.
- Die CT-Befunde bei Luftembolie sind der makromorphologischen Befunderhebung und -sicherung überlegen und somit additiv zur Obduktion erforderlich.
